# Sequential Treatment for Osteoporosis After Teriparatide: A Real-Life Long-Term Comparison Between Zoledronic Acid and Denosumab

**DOI:** 10.3390/jcm14186360

**Published:** 2025-09-09

**Authors:** Alberto Ghielmetti, Giorgia Grassi, Marta Zampogna, Giovanna Mantovani, Iacopo Chiodini, Cristina Eller-Vainicher

**Affiliations:** 1 Endocrine Unit, Fondazione IRCCS Ca’ Granda Ospedale Maggiore Policlinico, 20122 Milan, Italy; giorgia.grassi@policlinico.mi.it (G.G.); cristina.eller@policlinico.mi.it (C.E.-V.); 2 Department of Clinical Sciences and Community Health-Dipartimento di Eccellenza 2023–2027, University of Milan, 20122 Milan, Italy; 3 Department of Medical Biotechnology and Translational Medicine, University of Milan, 20100 Milan, Italy; 4 Unit of Endocrinology, ASST Grande Ospedale Metropolitano Niguarda, 20162 Milan, Italy

**Keywords:** osteoporosis, teriparatide, zoledronic acid, denosumab, sequential osteoporosis treatment

## Abstract

**Background/Objectives:** The efficacy of zoledronic acid (ZOL) compared to denosumab (DMAB) after teriparatide (TPTD) is largely unknown. We compared the effect of ZOL or DMAB treatment after TPTD on BMD changes and fracture (FX) occurrence. **Methods:** We retrospectively revised data from 77 patients treated at Fondazione IRCCS Ca’Granda Milan, Italy, with TPTD (≥18 months), given at withdrawal (T0), single ZOL 5 mg (Group A, N = 56) or DMAB 60 mg/6 months (Group B, N = 21). BMD changes and incident FX were assessed after 24 months (T1) in all patients and after 48 months (T2) in 46 patients (Group A1, N = 15, treated with a single ZOL at T0; Group A2, N = 17, treated with ZOL at T0 and T1; Group B, N = 14, treated with DMAB since T0 to T2). **Results:** During the T0–T1 period, in all groups, both spine (LS) and total hip (TH) T-scores improved (mean ± SD, T0 vs. T1): Group A (LS −2.5 ± 1.2 vs. −2.3 ± 1.3, *p* = 0.006; TH −2.2 ± 1.0 vs. −2.0 ± 1.1, *p* = 0.002) and Group B (LS −2.4 ± 1.4 vs. −1.8 ± 1.4, *p* < 0.001; TH −2.4 ± 1.0 vs. −2.2 ± 1.0, *p* = 0.003). At T2 vs. T0, all groups showed an increase in TH-BMD (A1 −1.8 ± 0.9 vs. −1.4 ± 1.0, *p* = 0.008; A2: −1.8 ± 0.8 vs. −1.6 ± 0.9, *p* = 0.032; B: −2.6 ± 0.7 vs. −2.2 ± 0.7, *p* < 0.001), while LS-BMD increased only in Group B (−2.7 ± 1.4 vs. −2.0 ± 1.2, *p* = 0.002), with stability in A1 and A2. No significant differences in incident FX between groups were observed. **Conclusions:** At 24 months after TPTD withdrawal, both ZOL and DMAB improved BMD at all sites; after 48 months, both ZOL (1 or 2 infusions) and DMAB led to BMD improvement at TH, whereas only DMAB led to an increase in LS-BMD.

## 1. Introduction

Osteoporosis is a systemic skeletal disorder characterized by low bone mass and micro-architectural deterioration of bone tissue, resulting in increased risk of fracture (FX) [1]. Currently, the availability of several anabolic and antiresorptive drugs aids in planning sequential therapy, aimed at reducing fracture risk [2,3]. In high-risk patients, anabolic therapy is indicated as the first intervention [2,3], considering that it can guarantee the most rapid and significant reduction in fracture risk, which is particularly recommended in patients at imminent risk of fracture (for instance, those having recently experienced a fragility fracture) [4]. However, upon discontinuation of anabolic drugs, in order to manage subsequent BMD loss, consolidation with an antiresorptive agent is required [2,3]. A second course of anabolic therapy with a different drug may be used in selected patients with residual high fracture risk [4].

One of the osteoanabolic agents approved for the treatment of osteoporosis is teriparatide (TPTD), i.e. parathyroid hormone (1-34), which, at the dose of 20 mcg/day, was proven to reduce vertebral and non-vertebral FX compared to placebo in postmenopausal women [5] and vertebral FX risk in postmenopausal patients with prior vertebral FX in comparison with risedronate [6]. It has to be added that TPTD efficacy, compared to placebo, has also been demonstrated in men in terms of BMD increase [7,8] and fracture incidence reduction [9]. Furthermore, dealing with one of the most common secondary causes of osteoporosis, in both men and women affected by glucocorticoid-induced osteoporosis, TPTD demonstrated superiority in BMD and vertebral FX incidence reduction as compared to alendronate after 18 months, with significant differences in LS BMD gain detectable from 6 months onwards [10]. Moreover, despite the fact anabolic treatment is recommended as the first intervention in patients with high fracture risk [2,3], it has been shown that transitioning from oral bisphosphonates to teriparatide (TPTD) results in greater BMD increases at LS as compared to transitioning to denosumab (DMAB) [11], a finding that should be taken into account in real-life clinical practice.

Nevertheless, significant BMD loss has been described after TPTD withdrawal [9,12,13,14,15], as its effects rapidly wear off. In this regard, in the setting of postmenopausal osteoporosis, Eastell et al. in the European Study of Forsteo (EUROFORS) described a mean 2.7% BMD loss at LS 12 months after TPTD interruption in the placebo arm, as compared to BMD stabilization at the lumbar spine and increase at the hip in women subsequently treated with raloxifene [13]. In the follow-up of Fracture Prevention Trial (FPT), without sequential anti-osteoporosis treatment, 18 months after TPTD withdrawal, approximately 40% of the BMD gains achieved with TPTD at LS were lost [14]. Similarly, Adami and colleagues experienced a 4% BMD loss at the lumbar spine (LS) in postmenopausal women 1 year after TPTD withdrawal, a phenomenon which, at variance with the findings of the EUROFORS study [13], was not prevented, although mitigated (mean BMD loss at LS 1%), with raloxifene [12]. Likewise, BMD loss after TPTD treatment not followed by bisphosphonates was observed also in men: BMD gradually decreased following discontinuation of TPTD therapy at LS and total hip, while subsequent antiresorptive treatment prevented the decline and tended to further increase BMD [9]. Interestingly, in the follow-up of FPT, vertebral fracture risk in women previously treated with TPTD 20 mcg/day and not subsequently treated with anti-osteoporosis drugs was not significantly different from that of women previously treated with placebo and subsequently not treated with any anti-osteoporosis drugs, although at multivariate analysis bisphosphonate use was not an independent predictor of vertebral fracture incidence after TPTD [14]. In the European Forsteo Observational Study (EFOS), involving women with severe postmenopausal osteoporosis, fracture risk decreased with TPTD, with no evidence of further change after TPTD was discontinued (18-month follow-up), but during this period, the majority of patients were under anti-osteoporosis medications and, mostly, bisphosphonates [16]. Therefore, an antiresorptive treatment is recommended after the completion of a course of TPTD treatment [2,3], which was approved for a maximum 24 months period [17], but there is no clear evidence on the best therapeutic strategy to follow.

Nevertheless, although the topic is extremely relevant from a clinical point of view, the available evidence is quite scarce, and only a few studies have explored the efficacy of antiresorptive treatment (bisphosphonates or DMAB) after TPTD. A treatment option after stopping TPTD is represented by denosumab (DMAB), which has proven to be superior to oral bisphosphonates (BPs) with regard to bone mineral density (BMD) gain [18], in particular at LS [19,20]. However, real-world data based on US prescription registries showed that only 40.8% of patients treated with TPTD switched, as recommended [2,3], to another treatment after withdrawal and that only 7% of these switched to intravenous BPs [21]. To the best of our knowledge, only two small real-life studies have focused on the efficacy of zoledronic acid (ZOL) treatment after TPTD withdrawal, in comparison with DMAB, showing beneficial effects at 12 months [22], and at 24 months [23], by both annual ZOL infusions and DMAB administration. Many aspects have to be considered and balanced when choosing the treatment sequence after the completion of osteoanabolic therapy. Reasonably, the optimal treatment at the end of TPTD should maintain the attained BMD targets and fracture risk in case of low fracture risk, while it should produce further BMD increases with a simultaneous reduction in fracture risk in the case of residual high fracture risk. Undoubtedly, cost-effectiveness, patient adherence, and required duration of treatment following osteoanabolic therapy are other relevant key points that should be considered in the choice of sequential antiresorptive therapy. In this regard, it should be specified that there is growing evidence that the antiresorptive efficacy of one ZOL administration can extend beyond one year, both in terms of BMD and reduction in fracture risk [24,25,26]. Moreover, ZOL, due to its intravenous and diluted way of administration, has proven to improve patient adherence to treatment compared to other bisphosphonates [27]. On the other hand, DMAB, unlike bisphosphonates, is characterized by a rapid cessation of its effects once interrupted, requiring sequential therapy with bisphosphonates, in order to avoid BMD loss and risk of multiple vertebral FX [28].

Given the absence of real-life long-term data on the effect of sequential therapy after TPTD withdrawal, the present study aimed primarily to describe BMD changes during the first 24 and 48 months after TPTD withdrawal, assessing the effects of the treatment with a single administration of ZOL 5 mg or with DMAB (conventional treatment 60 mg every 6 months). A secondary aim of the study was to evaluate the occurrence of incident vertebral and hip fragility FX during the first 48 months from TPTD withdrawal, in patients treated only with ZOL or only with DMAB during the entire follow-up period.

## 2. Materials and Methods

### 2.1. Subjects and Study Design

This retrospective study was conducted at the outpatient clinic for Metabolic Bone Diseases of Fondazione IRCCS Ca’ Granda Ospedale Maggiore Policlinico in Milan (Italy) and evaluated data of all osteoporotic patients treated with TPTD (20 mcg/day) from May 2009 to May 2023. We included data at baseline (T0), after 24 months (T1), and after 48 months (T2).

The inclusion criteria for a patient to be included in the T0–T1 evaluation were as follows: (i) TPTD treatment for ≥18 months (with ≥80% compliance); (ii) subsequent treatment with either ZOL 5 mg (single infusion) or DMAB (regularly administered 60 mg every 6 months), started within three months from TPTD withdrawal; (iii) availability of Dual-Energy X-ray Absorptiometry (DEXA) measurement at TPTD withdrawal and after 24 months from ZOL infusion or DMAB start performed with Hologic devices; and (iv) availability of spine X-ray to perform vertebral morphometry, at TPTD withdrawal and after 24 months from ZOL infusion or DMAB start. The exclusion criteria were as follows: (i) DMAB treatment duration of less than 24 months and/or unsatisfactory compliance with DMAB treatment (at least one injection delayed, ≥3 weeks, or missed) and (ii) DEXA measurements with different devices at the scheduled timepoints.

For a patient to be included in the T0–T2 evaluation, we used the following inclusion criteria: (i) treatment (period 0–48 months from TPTD withdrawal) with either ZOL 5 mg (at least one infusion) or DMAB (regularly administered 60 mg every 6 months for the entire follow-up period); (ii) availability of a Hologic DEXA measurement at T0 and T2; (iii) availability of spine X-ray at T0 and T2. Exclusion criteria were as follows: (i) unsatisfactory compliance with DMAB treatment (as previously defined); (ii) DEXA measurement with different devices at the scheduled timepoints (T0–T2); and (iii) switch from DMAB or ZOL to other anti-osteoporotic drugs during the follow-up period.

**Figure 1:**
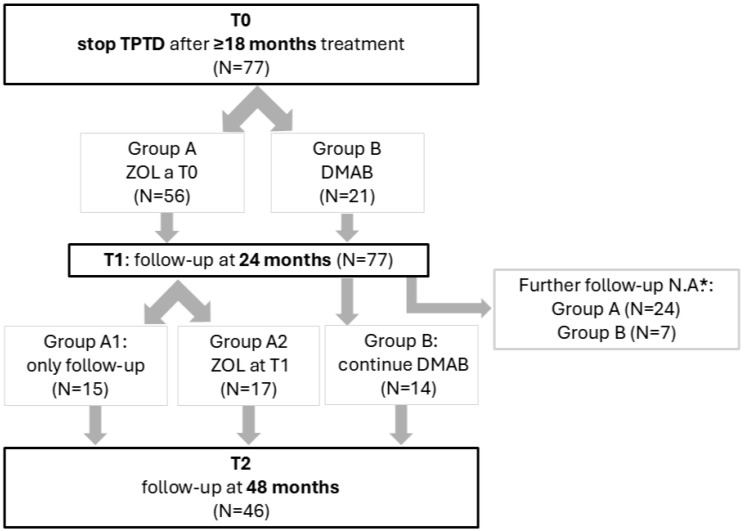
Summary of study design. TPTD = teriparatide; N.A. = not available because either absent or not conformable to inclusion/exclusion criteria for T2 evaluation, as specified in the Methods section. * In Group A after T1, 7 patients asked to be referred to other centers, 5 at the time of study analysis still had to come for T2 follow-up, 4 lacked a suitable T2 DEXA evaluation as defined in the Methods section, 4 were lost at the scheduled follow-up, 3 were shifted to DMAB for their convenience (possibility to administer therapy at home), and 1 died for reasons unrelated to osteoporosis (lung cancer developed after T1), whereas in Group B, 3 patients were shifted to bisphosphonates at T1 (having achieved a BMD target deemed satisfactory), 2 were lost at the scheduled follow-up, 1 was not compliant with DMAB therapy, and 1 lacked a suitable T2 DEXA evaluation as defined in the Methods section.

The study design, with division of patients into groups, is shown in Figure 1. At T0, we decided to treat patients with 1 dose of ZOL 5 mg (Group A) or with DMAB (Group B), according to good clinical judgement. After T1, a suitable follow-up (either absent or not conformable to inclusion/exclusion criteria) was not available in 31 patients; the reasons are detailed in the caption of Figure 1.

Among patients with further available follow-up at T2, some continued the follow-up after the first ZOL administration at T0 (Group A1), some repeated a second ZOL administration at T1 (Group A2), and some continued DMAB therapy (Group B). The 15 patients from Group A1 did not undergo a second ZOL infusion because either they had a T-score > −2.5 at all sites (N = 7), refused other anti-osteoporotic treatments (N = 3), or, despite having regularly performed at T1 DEXA and spine X-rays, were lost at the scheduled follow-up visit at 24 months due to pandemic-related reasons (N = 5).

Ethical approval was obtained from the Ethics Committee (Milan, Lombardia 3) (ID 5165, response 17 September 2024).

### 2.2. Methods

For all subjects at T0, the following data were collected: age, gender, type of osteoporosis (primary or secondary), use of antiresorptive therapy before TPTD and its duration, duration of TPTD treatment, prevalent and incident fragility FX, 10-year risk for major osteoporotic FX, hip FX estimated using the FRAX Calculation Tool [29], serum total calcium levels, and serum creatinine levels. All patients took adequate vitamin D and calcium supplements during the entire study duration, if needed.

The BMD measurements at the lumbar spine (LS) and total hip (TH) were performed using DEXA (Hologic systems). The BMD measurements were expressed as T-scores, which were calculated by the third National Health and Nutrition Examination Survey, using data from young White females as a reference [30].

At T0, T1, and T2, a conventional spine X-ray in lateral and anteroposterior projection (T4–L4) was obtained in all patients using a standardized technique. Morphometric Vertebral Fractures (FX) were identified according to the semiquantitative visual assessment (SQ) [31]. Fractures were defined as a reduction of >20% in anterior, middle, or posterior vertebral height. From lateral spine radiographs, 13 vertebrae from T4 to L4 were assessed visually as intact (SQ grade 0) or as having approximately mild (20% to 25% height reduction), moderate (25% to 40% height reduction), or severe (>40% height reduction) deformity (SQ grades 1, 2, and 3, respectively). In all patients, we also evaluated the spinal deformity index (SDI), calculated by summing the fracture grades of all vertebrae (T4 to L4), a method used for predicting future vertebral FX risk [32]. Two radiologists, who were unaware of the BMD data, independently reviewed the radiographs. The questionable cases were collectively discussed to agree on a diagnosis. Serum calcium and creatinine levels were measured using commercially available assay kits from outpatient diagnostic sources.

### 2.3. Statistical Analysis

For each variable, the normality of distribution was tested using the Kolmogorov–Smirnov test. Quantitative variables were expressed as the mean ± standard deviation or median (interquartile range) when not normally distributed. Comparisons between two groups (A and B) were made using independent sample *t*-tests or Mann–Whitney U tests, as appropriate. Comparisons within two groups at T0 and T1 timepoints were made using paired samples *t*-tests or the paired samples Wilcoxon test, as appropriate. The comparisons between three groups (A1, A2, and B) were made via one-way ANOVA with Bonferroni post hoc tests or the Kruskal–Wallis test for independent samples and post hoc pairwise comparisons with Bonferroni correction for multiple tests, as appropriate. The comparisons within three groups at T0, T1, and T2 timepoints were made using Repeated Measures ANOVA or Friedman’s ANOVA and post hoc pairwise comparisons with Bonferroni correction for multiple tests, as appropriate. Categorical variables were expressed as an absolute number count (percentage) and compared with chi-squared tests or Fisher’s exact tests, as appropriate. Statistical analyses were performed using SPSS version 28.0 statistical package software (SPSS Inc., Chicago, IL, USA).

The BMD gain is expressed as median ΔT-score, as follows.

Two-tailed *p*-values < 0.05 were considered statistically significant.

## 3. Results

### 3.1. BMD Changes

Clinical and demographic characteristics at T0 of all included patients and patients included in Group A and Group B are shown in Table 1. The two groups were comparable in terms of age, gender distribution, prevalence of secondary osteoporosis, use of antiresorptive agents (ARs) prior to TPTD, years of ARs use prior to TPTD, months of TPTD treatment, prevalence of vertebral and hip FX, SDI, FRAX score, BMD at LS and TH, serum calcium, and creatinine.

**Figure 2:**
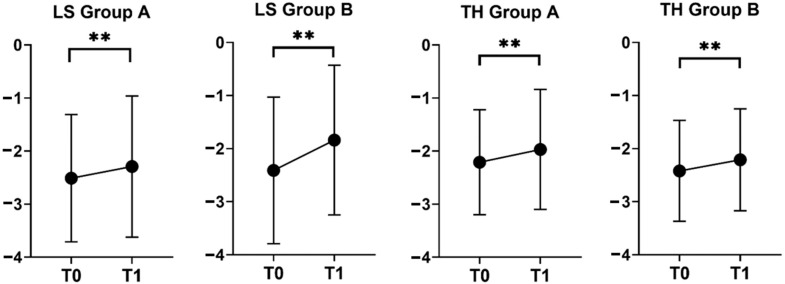
BMD trends in groups A (treated with ZOL at T0, N = 56) and B (treated with DMAB, N = 21). Data are expressed as the mean T-score ± SD. ** *p* < 0.01.

**Figure 3:**
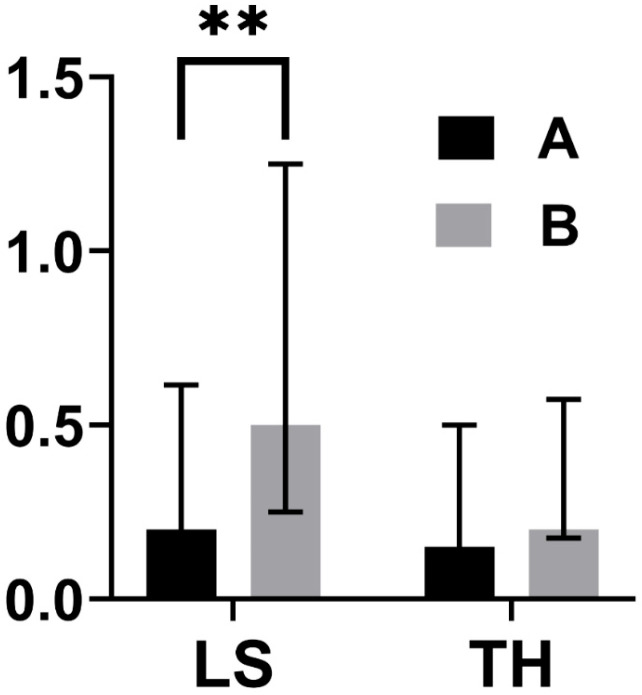
Differences between ΔT-score (T1 − T0), expressed as median (interquartile range), in groups A (treated with ZOL at T0, N = 56) and B (treated with DMAB, N = 21). ** *p* < 0.01.

Considering both groups A and B, a significant improvement in T-score was noticed at all sites in the T0–T1 period (Table 2; Figure 2), with median T-score increase at LS being higher in Group B as compared to Group A (Table 3; Figure 3).

In the subgroup of 46 patients with available follow-up at both 24 and 48 months (T1 and T2), we compared the baseline (T0) characteristics of the A1, A2, and B groups. We found that patients in Group A2 had experienced a shorter duration of TPTD treatment than those in Group A1 (18.3 (3.6) vs. 24.3 (0.4) months, *p* < 0.001) and that the T-score at TH was lower in Group B than both groups A1 and A2 (Table 4).

Comparisons between groups A1, A2, and B at T0, T1, and T2 timepoints are given in Table 4 and Table 5. In all groups, the TH T-score at T2 improved as compared to T0, whereas the LS T-score improved only in Group B and remained stable in groups A1 and A2 (Table 4). No significant differences in median T-score increase in BMD within groups at different timepoints, nor between groups, were noticed (Table 5).

### 3.2. Incidence of FX

During the first 24 months of observation, three patients suffered from an incident vertebral FX (3/77, 3.9%), and they all belonged to Group A (5.3 vs. 0.0%, *p* = 0.28), while no hip FX occurred. Hip BMD values at T0 were worse in patients with incident vertebral FX (TH −3.5 ± 0.4) as compared to those without incident FX (TH −2.2 ± 0.9, *p* = 0.028); moreover, patients with incident FX tended to have received a shorter treatment with TPTD (median 18 vs. 24 months, *p* = 0.062) than patients without incident FX. No incident vertebral or hip FX were observed during the period T1–T2.

## 4. Discussion

This real-life study provides novel and clinically relevant evidence showing that the initiation of antiresorptive treatment immediately after the completion of teriparatide (TPTD) therapy can lead to substantial and measurable improvements in bone mineral density (BMD). These positive effects were observed both at the lumbar spine (LS) and at the total hip (TH) after a 24-month follow-up, and they were consistently present regardless of whether patients received a single intravenous infusion of zoledronic acid (ZOL) or repeated subcutaneous administrations of denosumab (DMAB). It is important to underline that, in our cohort, the improvements observed at the hip site were not only preserved but remained stable for as long as 48 months in the group treated with ZOL. In contrast, patients who were managed with DMAB displayed a continuous and progressive increase in BMD at all skeletal sites assessed, confirming the well-recognized and persistent antiresorptive action of this therapeutic agent, which proved to ensure a sustained BMD gain for up to 10 years, without reaching a plateau [33].

To the best of our knowledge, this study represents the first real-world investigation demonstrating that a single infusion of ZOL at the conventional dose of 5 mg, administered at the time of TPTD withdrawal, is capable not only of maintaining the BMD previously gained but also of further enhancing it, particularly at the hip level, with effects lasting for as long as 48 months. This observation stands in contrast to the majority of earlier studies, which generally reported stabilization of BMD with annual ZOL administrations observed at 12 and 24 months [22,23]. Our results therefore suggest that ZOL may exert an even stronger and more durable effect when given immediately after the discontinuation of anabolic therapy with TPTD. This interpretation is supported by evidence, recently provided by Giveon and colleagues, that a single ZOL infusion in this context is associated with a deeper and more prolonged suppression of serum C-terminal telopeptide (CTX), an effect that can persist for up to four years, compared with patients treated with ZOL alone, irrespective of the duration of prior oral bisphosphonate exposure [34]. This enhanced efficacy is likely related to the increased bone turnover induced by TPTD, a condition in which antiresorptive therapy may act more efficiently. In line with this, BMD gain at the lumbar spine (LS) has been shown to be directly associated with serum procollagen type 1 N-terminal propeptide (P1NP) levels measured at the moment of TPTD withdrawal [20]. Also, considering the known long-lasting antiresorptive action of ZOL, which may extend well beyond 12 months [24,25,26], its use appears particularly justified in patients with a relatively low residual fracture risk who can benefit from a simple and durable therapeutic approach. In fact, in osteoporotic patients, data from the HORIZON trials show that the antifracture efficacy of ZOL can last for more than 12 months, with it being comparable at 3 years in the case of single administration or conventional annual administration [24]. Likewise, in osteopenic women, a single 5 mg ZOL administration can prevent BMD loss for up to 10 years and reduce serum CTX levels for 9 years [25]. Finally, it has recently been described that in women with normal BMD or those who are osteopenic, ZOL 5 mg administered every 5 years results in a significant reduction in incident vertebral FX during a 10-year follow-up [26].

In our study, while both DMAB and ZOL were able to produce significant BMD increases at the 24-month evaluation, DMAB administration resulted in a significantly greater gain at LS. This result is consistent with previous findings showing that DMAB is particularly effective in trabecular-rich skeletal sites, an effect that has been observed in sequential treatment regimens following TPTD when compared to oral bisphosphonates [18,19] and to bisphosphonates in general, which were mainly administered orally [20]. On the other hand, ZOL demonstrated its ability to improve TH-BMD with only a single infusion, a result that, to our knowledge, had not been clearly reported before. Furthermore, our observations expand upon the findings from the HORIZON trials and from studies on romosozumab transitions [24,35], which showed that even non-repeated ZOL administrations can sustain BMD benefits over time. Indeed, a single dose of ZOL 5 mg has been shown to increase hip BMD after three years [24] and maintain the stability of BMD at all skeletal sites after two years when given following anabolic treatment with romosozumab [35]. Although our results should be interpreted with caution due to the limited sample size of our cohort, it is worth noting that, in accordance with our findings, after TPTD therapy, ZOL administration has already been associated with a larger BMD increase at the hip when compared with oral bisphosphonates [20].

From a clinical management point of view, the use of a single ZOL infusion has additional advantages beyond efficacy. The simplicity of the regimen, requiring only one administration, may significantly enhance patient adherence, which is an essential determinant of therapeutic success [27,36]. This approach also reduces the need for frequent clinical monitoring and can represent a cost-effective option in healthcare systems with limited resources. Such aspects are particularly relevant in older or frail patient populations, where the logistics of regular six-monthly DMAB injections or repeated annual infusions of ZOL may present significant challenges. It should also be remembered that poor adherence to DMAB therapy can lead to dramatic consequences, including substantial BMD loss and an increased risk of vertebral fractures that can nullify all of the results achieved [28]. These practical considerations strongly warrant further evaluation of the long-term cost-effectiveness and real-world feasibility of single-infusion ZOL strategies [24,25].

Regarding fracture outcomes, our study did not detect a statistically significant difference in the incidence of vertebral or hip fractures between the two therapeutic groups, a finding that is consistent with those of previous real-life studies [23]. Nevertheless, it is noteworthy that all incident vertebral FX occurred in the ZOL group, and these cases were characterized by lower baseline TH-BMD values and a shorter exposure to TPTD. These observations underline the importance of completing the full 24-month course of TPTD therapy whenever possible [37], as well as carefully evaluating hip BMD and residual fracture risk before selecting the most appropriate consolidation strategy. It has been demonstrated that increases in hip BMD under ZOL treatment are closely associated with reductions in vertebral fracture risk [38], reinforcing the role of this site as a key determinant in clinical decision-making.

The potential incorporation of bone turnover markers (BTMs) into clinical practice could provide an additional tool for optimizing post-anabolic therapy. Markers such as serum CTX or P1NP have been proposed to help identify patients at higher risk of early bone loss, thereby allowing clinicians to individualize the timing and possible repetition of ZOL administration [39,40]. Indeed, strategies guided by BTMs have already demonstrated promising results, showing that bisphosphonate re-dosing based on marker levels can maintain BMD and reduce fracture incidence while minimizing the number of infusions required. In one study, only 39% of patients needed more than a single infusion during a median four-year follow-up when a marker-driven approach was applied [40]. This highlights the potential of BTMs to personalize sequential treatment regimens, reduce overtreatment, and optimize patient outcomes.

Our study, however, presents several limitations that should be acknowledged. First, the retrospective and non-randomized design means that treatment allocation—whether ZOL or DMAB—was based on clinical judgment rather than on standardized protocols, and the decision to repeat ZOL infusions was similarly individualized. Although baseline characteristics were comparable across the study groups, the possibility of unmeasured confounding factors cannot be excluded. Second, the relatively small sample size, particularly in the subgroup of patients followed for 48 months, may have reduced the statistical power to detect significant differences in fracture incidence. Finally, we did not have access to BTMs in our dataset, which would have provided valuable additional insights into treatment response and might have helped to further personalize post-anabolic strategies. Future prospective studies with randomized allocation and systematic incorporation of BTMs are therefore warranted to validate and expand our observations.

In conclusion, our findings indicate that both ZOL and DMAB can be considered effective consolidation strategies following TPTD withdrawal, but their optimal use should be carefully tailored to the individual patient’s characteristics and residual fracture risk. In those with low residual risk, a single infusion of ZOL seems to be sufficient to maintain the benefits achieved, particularly by sustaining hip BMD gains and preventing lumbar spine BMD loss for up to 48 months, while offering the additional advantage of simplicity and improved adherence. In contrast, in patients with persistently high fracture risk, DMAB may represent the preferred option, especially given its superior efficacy at the lumbar spine, with the essential caveat that discontinuation of DMAB must always be accompanied by bisphosphonate administration to avoid the severe rebound effect characterized by rapid BMD loss and multiple vertebral fractures [28,41,42]. Another therapeutic alternative for patients at high risk, although not available in Italy during the period of this study, is represented by transitioning from TPTD to romosozumab [43], a sequence that combines anabolic and antiresorptive properties and could potentially maximize skeletal benefits.

Overall, these real-world results strongly emphasize the need for prospective randomized trials specifically designed to assess long-term and personalized treatment strategies after TPTD therapy. Such trials should take into account fracture risk stratification, BMD profiles, duration of anabolic treatment, and possibly BTMs, with the ultimate goal of developing individualized algorithms capable of optimizing both clinical outcomes and healthcare resource utilization in the long-term management of osteoporosis.

## Figures and Tables

**Table 1:** Clinical and demographic characteristics at T0 in all patients and in groups A and B.

Variables at T0	All (N = 77)	Group A (N = 56)	Group B (N = 21)	P
Age (years)	71.1 (15.9)	67.9 (16.6)	74.0 (11.7)	0.185
Female gender	64 (83.1)	46 (82.1)	18 (85.7)	0.709
Secondary OP	37 (48.0)	29 (51.8)	8 (38.1)	0.317
ARs prior to TPTD	65 (84.4)	49 (87.5)	16 (76.2)	0.291
Years of ARs prior to TPTD	4.0 (6.5)	4.5 (5.5)	3.0 (8.5)	0.648
TPTD treatment (months)	24 (5.1)	23.9 (6.1)	24.1 (0.35)	0.140
Vertebral FX (≥1)	77 (100)	56 (100)	21 (100)	1.000
SDI	7 (7)	7 (9)	9 (6)	0.153
Hip FX (≥1)	8 (10.4)	5 (8.9)	3 (14.3)	0.676
FRAX (10 years major fracture risk, %)	28.5 (21.3)	27 (19.3)	30.5 (27)	0.143
FRAX (10 years hip fracture risk, %)	12.5 (16.1)	11 (14.9)	14 (23.3)	0.162
T-score TH	−2.26 ± 0.98	−2.21 ± 0.99	−2.42 ± 0.95	0.251
T-score LS	−2.48 ± 1.25	−2.51 ± 1.20	−2.41 ± 1.38	0.320
Serum calcium (mg/dL)	9.54 ± 0.42	9.50 ± 0.39	9.65 ± 0.46	0.217
Serum creatinine (mg/dL)	0.81 (0.29)	0.81 (0.32)	0.81 (0.35)	0.495

Group A: patients treated with Zoledronate; Group B: patients treated with denosumab. OP: osteoporosis; ARs: antiresorptive agents; TPTD: teriparatide; FX: fractures; SDI: Spine Deformity Index; TH: total hip; LS: lumbar spine; calcium normal values 8.1–10.5 mg/dL; creatinine normal values 0.6–1.2 mg/dL.

**Table 2:** BMD trends in groups A and B (period T0–T1).

Variable	Group A T0 (N = 56)	Group A T1 (N = 56)	P	Group B T0 (N = 21)	Group B T1 (N = 21)	P
T-score TH	−2.21 ± 0.99	−1.97 ± 1.13	0.002	−2.42 ± 0.95	−2.21 ± 0.96	0.001
T-score LS	−2.51 ± 1.20	−2.29 ± 1.33	0.006	−2.41 ± 1.38	−1.84 ± 1.41	<0.001

Group A: patients treated with Zoledronate; Group B: patients treated with denosumab; TH: total hip; LS: lumbar spine.

**Table 3:** BMD gains in groups A and B (period T0–T1).

Variable	Group A (N = 56)	Group B (N = 21)	P
ΔT TH (T1 − T0)	0.15 (0.40)	0.20 (0.35)	0.740
ΔT LS (T1 − T0)	0.20 (0.43)	0.50 (0.50)	0.004

Group A: patients treated with Zoledronate; Group B: patients treated with denosumab; TH: total hip; LS: lumbar spine; ΔT = ∆T-score, expressed as median (interquartile range).

**Table 4:** Comparisons within and between groups A1, A2, and B in patients with available follow-up at T0, T1, and T2 timepoints (Group A has been split into A1 and A2, also at T0 and T1).

Variable	Group A1 T0 (N = 15)	Group A1 T1 (N = 15)	Group A1 T2 (N = 15)	P Within A1	Group A2 T0 (N = 17)	Group A2 T1 (N =17)	Group A2 T2 (N = 17)	P Within A2	Group B T0 (N = 14)	Group B T1 (N = 14)	Group B T2 (N = 14)	P Within B	P Between Groups
T-score TH	−1.78 ± 0.87	−1.49 ± 0.86	−1.44 ± 0.98	0.004 *	−1.78 ± 0.79	−1.58 ± 0.85	−1.57 ± 0.93	0.008 §	−2.57 ± 0.72	−2.32 ± 0.77	−2.19 ± 0.68	<0.001 #	T0: 0.030 ^ T1: 0.034 $ T2: 0.082
T-score LS	−2.21 ± 1.17	−1.94 ± 1.22	−2.13 ± 1.54	0.041 +	−1.99 ± 1.17	−1.87 ± 1.40	−1.73 ± 1.43	0.203	−2.67 ± 1.40	−2.17 ± 1.38	−2.02 ± 1.25	<0.001 °	T0: 0.400 T1: 0.992 T2: 0.583

TH: total hip; LS: lumbar spine. From post hoc comparisons of within-groups tests: * T1 vs. T0 *p* = 0.006, T2 vs. T0 *p* = 0.004; § T1 vs. T0 *p* = 0.022, T2 vs. T0 *p* = 0.013; # T1 vs. T0 *p* = 0.008, T2 vs. T0 *p* < 0.001; + T1 vs. T0 *p* = 0.014; ° T1 vs. T0 *p* = 0.006; T2 vs. T0 *p* < 0.001. From post hoc comparisons of between-groups tests: ^ B vs. A1 *p* = 0.028, B vs. A2 *p* = 0.017; $ B vs. A1 *p* = 0.028, B vs. A2 *p* = 0.020.

**Table 5:** Comparisons within and between groups A1, A2, and B in patients with available follow-up at T0, T1, and T2 timepoints (Group A has been split into A1 and A2, also at T0 and T1).

Median ΔT-Score	Group A1 T0T1 (N = 15)	Group A1 T1T2 (N = 15)	Group A1 T0T2 (N = 15)	P Within A1 ^1^	Group A2 T0T1 (N = 17)	Group A2 T1T2 (N = 17)	Group A2 T0T2 (N = 17)	P Within A2 ^1^	Group B T0T1 (N = 14)	Group B T1T2 (N = 14)	Group B T0T2 (N = 14)	P Within B ^1^	P Between Groups
TH	0.3 (0.2)	0.1 (0.5)	0.3 (0.5)	0.120	0.1 (0.4)	0 (0.3)	0.3 (0.5)	0.139	0.2(0.4)	0.1 (0.5)	0.3 (0.3)	0.360	T0T1: 0.182 T1T2: 0.275 T0T2: 0.167
LS	0.3 (0.3)	−0.2 (0.9)	0.2 (1.3)	0.109	0.2 (0.8)	0.1 (0.4)	0.3 (0.8)	0.820	0.5 (0.5)	0.1 (0.8)	0.6 (0.4)	0.410	T0T1: 0.607 T1T2: 0.487 T0T2: 0.547

TH: total hip; LS: lumbar spine. ^1^ Paired comparisons between T0T1 and T1T2.

## Data Availability

The Dataset is available from the authors upon request.

## Abbreviations

The following abbreviations are used in this manuscript:

ANOVA	Analysis of Variance
BMD	Bone mineral density
BTMs	Bone Turnover Markers
CTX	C-terminal telopeptide
DEXA	Dual-Energy X-ray Absorptiometry
DMAB	Denosumab
EUROFORS	European Study of Forsteo
FPT	Fracture Prevention Trial
FX	Fracture(s)
LS	Lumbar Spine
P1NP	Procollagen Type 1 N-terminal Propeptide
SD	Standard Deviation
SDI	Spine Deformity Index
SQ	Semi-quantitative
TH	Total hip
TPTD	Teriparatide
US	United States
ZOL	Zoledronic acid
